# 2L polyethylene glycol combined with castor oil versus 4L polyethylene glycol for bowel preparation before colonoscopy among inpatients

**DOI:** 10.1097/MD.0000000000034294

**Published:** 2023-07-21

**Authors:** Zhe Xiong, Ying Fang, Fangfang Feng, Yiming Cheng, Chunyan Huo, Jin Huang

**Affiliations:** a Department of Gastroenterology, Changzhou Second People’s Hospital of Nanjing Medical University, Changzhou, China; b Department of Gastroenterology, Dalian Medical University, Dalian, Liaoning, China.

**Keywords:** bowel preparation, castor oil, colonoscopy, inpatients, polyethylene glycol

## Abstract

Inpatients are more likely to have inadequate bowel preparation compared to outpatients. Although experts recommend 4L split polyethylene glycol (PEG) preparation, bowel preparation with castor oil (CaO) was recently found to reduce the volume of solution required. The aim of the study was to evaluate the cleansing effect and safety of 2L-PEG with Cao in bowel preparation among inpatients.

Our study retrospectively analyzed the medical records and colonoscopy reports of inpatients (n = 1251) who underwent colonoscopy in the Affiliated Changzhou No.2 People Hospital of Nanjing Medical University, and the inpatients were divided into 2L-PEG-CaO and 4L-PEG group according to different bowel preparation protocols. Boston Bowel Preparation Scale (BBPS) is used to assess bowel preparation efficacy before colonoscopy. Furthermore, we also calculated other outcomes, such as polyp or adenoma detection rates and adverse events. A total of 1251 patients undergoing colonoscopy were included in this study, 738 were taken 4L-PEG and 513 2L-PEG-CaO. Both inpatients groups were matched for baseline characteristics. The 2L-PEG-CaO group was significantly higher than the 4L-PEG group on both BBPS (7.26 ± 1.75 vs 7.06 ± 1.58, *P* = .043) and adequate bowel cleansing rates (83.2% vs 77.4%, *P* = .011). Regarding adverse events, the 4L-PEG group was significantly higher than the 2L-PEG-CaO group on the incidence of abdominal fullness (6.4% vs 9.6%, *P* = .045) and adverse events (33.7% vs 28.5%, *P* = .048). The 2L split PEG with CaO preparation increased quality of bowel cleansing and improved tolerance in inpatients. Bowel preparation with 2L-PEG-CaO is suitable alternative to traditional 4L split PEG bowel preparation for colonoscopy of inpatients.

## 1. Introduction

The incidence of colorectal cancer, one of the most common cancers worldwide, has recently shown an upward trend in Asian countries, posing a huge burden on healthcare.^[1]^ Colonoscopy is the most effective method for early diagnosis and prevention of colorectal cancer.^[2]^ As is known to all, adequate bowel preparation is a key part of colonoscopy as it effectively improves the diagnostic performance of the test.^[3–5]^ Inadequate bowel preparation often results in increased missed diagnosis of precancerous and cancerous lesions, prolonged procedure time, suboptimal cecum insertion rates, and increased risk of complications.^[6]^

Currently, inpatient status has been identified as an important risk factor for inadequate colon cleansing, and in fact about 50% to 70% of inpatients achieving a competent colon cleansing, which is still a long way from our imagined threshold of 90%.^[7,8]^ In addition, inpatients are often considered to be “hard-to-prepare,”^[9]^ which may be related to the fact that inpatients have more severe disease than outpatients.^[10]^ Therefore, researchers have recently been investigating ways to improve bowel cleansing in inpatients, such as face-to-face instruction for patients with risk factors for inadequate bowel preparation^[11]^ or changes in the dose of medications taken. To date, polyethylene glycol (PEG) solutions have become the most common protocol before colonoscopy.^[12]^ However, standard 4L high volumes of PEG reduce patient tolerance and compliance.^[13]^ Therefore, to reduce the volume of fluid required, researchers combine PEG with ascorbic acid or bisacodyl to improve inpatient compliance.^[12,14]^ Even so, this is still a long way from the quality we expect from bowel preparation, it is therefore always worth exploring how to improve the quality of bowel preparation prior to colonoscopy in inpatients.

There is still some controversy regarding studies on reducing the volume of fluid in standard 4L PEG in order to reduce the amount of fluid consumed by patients to increase patient tolerance. A randomized controlled study found that 4L PEG was superior to 36 mg senna and 2L PEG as bowel preparation before colonoscopy.^[15]^ But there are also studies that show that 2L PEG combined with bisacodyl^[16]^ or combined with sodium phosphate^[17]^ also achieves the ideal bowel cleansing effect, reduces the volume of fluid required and increases patients tolerance and willingness to repeat the preparation when compared to the standard 4L PEG regimen. Castor oil (CaO) as a safe and effective stimulant laxative has been proved to be very effective in colon cleansing. For example, Yang et al^[18]^ suggested that CaO and bisacodyl were comparable in their laxative efficacy. Moreover, a multicenter study found that bowel preparations with CaO increased the capsule excretion rate and reduced liquid loading.^[19]^ However, there are few studies exploring the cleansing effects of CaO in bowel preparation before colonoscopy.

The aims of this study was to evaluate the effectiveness and patients tolerability of the 2L split PEG solution plus CaO preparation among inpatients, and to compare it with the standard 4L split PEG solution preparation with the aim of finding a better regimen of bowel preparation.

## 2. Materials and methods

### 2.1. Data collection

We conducted a retrospective cohort analysis of data collected form inpatients with underwent colonoscopy at the Affiliated Changzhou No.2 People Hospital of Nanjing Medical University from June 2020 to December 2021. According to the different bowel preparation formulations, we divided the inpatients into 4L-PEG group and 2L-PEG-CaO group. Meanwhile, we collected medical information of inpatients from the hospital electronic case system, including age, gender, height, weight, BMI, reason for colonoscopy, comorbidities, sedation medication, dosing of PEG (Taking ≥ 80% of the overall formulation indicates good patient compliance), Boston Bowel Preparation Scale (BBPS), adverse events during bowel preparation and colonoscopy reports. The study was conducted under the Helsinki Declaration. All patients signed informed consent for the procedure. This work was approved by the Ethics Committee of Changzhou Second People Hospital affiliated with Nanjing Medical University.

### 2.2. Patients

Adult inpatients who underwent colonoscopy in the morning for any indication and had a complete medical history were included in this study, whereas inpatients who did not complete bowel preparation due to their severe disease or drug allergy and those who underwent polypectomy, endoscopic mucosal resection, and endoscopic submucosal dissection were excluded.

### 2.3. Bowel preparation

All patients received intensive bowel preparation instructions for bowel preparation during hospitalization. Briefly, patients will receive guidelines for bowel preparation and they will be guided by the ward nurse or doctor on how to prepare their bowel before colonoscopy. All patients undergoing colonoscopy in the morning were asked to eat a low-fiber diet the day before and to start fasting at about 20:00 the night before. To improve tolerance and reduce the incidence of hypoglycemia, patients were allowed to eat sugar and bread during bowel preparation. The patients in the 2L-PEG-CaO group were asked to take 40 mL of CaO at 8:00 pm the day before colonoscopy; then, they were asked to take 2L PEG solution (each liter containing 64g PEG 4000, 1.46g sodium chloride, 5.7g sodium sulfate, 0.75g potassium chloride, 1.68g sodium bicarbonate) 4 to 6 hours before colonoscopy. The patients in the 4L-PEG group were asked to begin to drink the first 2L of PEG solution at 8:00 pm the day before colonoscopy; then, they took the other 2L PEG solution 4 to 6 hours before colonoscopy. The patients were guided to consume 250 mL of PEG solution every 15 minutes, and the preparation should be done 2 hours before colonoscopy.

### 2.4. Primary outcome

Colonoscopies of all patients were performed by the 5 senior endoscopists at the Gastroenterology Centre, each of them had performed at least 1000 colonoscopies. They assessed bowel cleanliness in segments based on the Boston Bowel Preparedness Scale (BBPS)^[20]^: 0 = More solid or semi-solid stool remains, intestinal mucosa not clearly visible; 1 = Some of the bowel mucosa is clearly visible, most of it is still not clearly visible due to staining, fecal masses or opaque mucus residue; 2 = A small amount of staining, granular stool or opaque mucous residue, but does not affect the clear exposure of intestinal mucosa; 3 = No staining, fecal or opaque mucus residue, the intestinal mucosa is completely visible. Segmental method: right colon (cecum to ascending colon), transverse colon (including hepatic and splenic flexures), left colon (descending colon and rectum). All bowel segment scores were added together for a total score, with higher scores indicating better quality bowel preparation. Adequate bowel preparation was defined as a total BBPS score was ≥6, and a score was ≥2 for each colon segment. High quality bowel preparation was defined as BBPS = 3 for each colonic segment and total score = 9.

### 2.5. Secondary outcomes

We calculated detection rate of polyp and adenoma (the proportion of polyps and adenomas detected during colonoscopy) and adverse events (e.g., abdominal fullness, abdominal pain, nausea, vomiting) from the nursing records and colonoscopy reports, and defined them as secondary outcomes.

### 2.6. Statistical analysis

SPSS 25.0 for Windows (SPSS, Chicago, IL) was used to analyze all data. Continuous variables were expressed as mean ± standard deviation, and discontinuous variables were expressed as counts and percentages. Comparisons between groups were made using the Student *t* test, data for categorical variables were tested using chi-square or Fisher exact test. The *P* values < .05 were considered statistically significant.

## 3. Results

### 3.1. Baseline characteristics

We consulted case information from 1251 patients, and according to the different bowel preparation formulations, we divided the inpatients into 4L-PEG group and 2L-PEG-CaO group. We divided patients into 2 groups, 738 patients received 4L PEG and 513 patients received 2L PEG plus 40 mL CaO. There were no significant differences in baseline characteristics between the 2 groups in terms of age, gender, weight, BMI, reason for colonoscopy, comorbidities, sedation medication and dosing of PEG (*P* > .05) (Table 1).

**Table 1:** Characteristics of the patients.

	2-PEG-CaO (n = 513)	4-PEG (n = 738)	*P* value
Age (mean ± SD, yr)	56.12 ± 13.06	56.73 ± 12.26	.394
Sex, n (%)			.231
Male	266 (51.9)	408 (55.3)	
Female	247 (48.1)	330 (44.7)	
Height (mean ± SD, cm)	165.33 ± 7.26	165.51 ± 7.16	.661
Weight (mean ± SD, kg)	63.14 ± 10.47	62.54 ± 11.30	.342
BMI (mean ± SD, kg)	23.02 ± 2.90	22.74 ± 3.35	.118
Non-sedation colonoscopy, n (%)	4 (0.8)	7 (0.9)	.753
Sedation colonoscopy, n (%)	509 (99.2)	731 (99.1)	.753
Compliance to preparation (≥80% intake), n (%)	491 (95.7)	689 (93.4)	.077
Medical condition, n (%)			
Hypertension	184 (35.9)	290 (39.3)	.219
Diabetes mellitus	119 (23.2)	181 (24.5)	.588
Previous abdominal surgery	57 (11.1)	69 (9.3)	.309
Cardiovascular disease	14 (2.7)	23 (3.1)	.691
Indication for colonoscopy, n (%)			
Bleeding rectum	114 (22.2)	146 (19.8)	.296
Abdominal pain	139 (27.1)	226 (30.6)	.177
Diarrhea	44 (8.6)	69 (9.3)	.639
Constipation	32 (6.2)	31 (4.2)	.105
Occult blood positive	6 (1.2)	11 (1.5)	.630
Anemia	35 (6.8)	64 (8.7)	.233
Screening/Surveillance	152 (29.6)	203 (27.5)	.413
Change in bowel habits	14 (2.7)	28 (3.8)	.304

CaO = castor oil, PEG = polyethylene glycol, SD = standard deviation.

### 3.2. Primary outcome

In this study, the 2L-PEG-Cao group was significantly higher than the 4L-PEG group on both BBPS (7.26 ± 1.75 vs 7.06 ± 1.58, *P* = .043) and adequate bowel cleansing rates (83.2% vs 77.4%, *P* = .011). At the same time, the 2L-PEG-CaO group had a significantly higher proportion of inpatients completing high-quality bowel preparation than the 4L-PEG group (34.7% vs 21.4%, *P* < .001). The analysis of the segmental (right, transverse, and left colon) BBPS scale showed no difference for the transverse side (2.41 ± 0.68 vs 2.35 ± 0.67) and left colon (2.48 ± 0.66 vs 2.43 ± 0.60). However, the right colon score was significantly higher in the 2L-PEG-CaO group than in the 4L-PEG group (2.37 ± 0.69 vs 2.28 ± 0.75, *P* = .034). Table 2 presents the results of bowel cleansing quality assessment based on the BBPS.

**Table 2:** Comparison of Boston bowel preparation score between the 2 groups.

	2-PEG-CaO (n = 513)	4-PEG (n = 738)	*P* value
Adequate colon cleansing, n (%)	427 (83.2)	571 (77.4)	.011*
High quality colon cleansing, n (%)	178 (34.7)	158 (21.4)	<.001*
Right side of colon (mean ± SD)	2.37 ± 0.69	2.28 ± 0.75	.034*
Mid colon (mean ± SD)	2.41 ± 0.68	2.35 ± 0.67	.136
Left side of colon (mean ± SD)	2.48 ± 0.66	2.43 ± 0.60	.163
BBPS (mean ± SD)	7.26 ± 1.75	7.06 ± 1.58	.043*

BBPS = Boston Bowel Preparation Scale, CaO = castor oil, PEG = polyethylene glycol, SD = standard deviation.

* *P* < .05.

### 3.3. Secondary outcome

The rates of polyp detection in the 2L-PEG-CaO and 4L-PEG group were 10.3% and 11.5%, adenoma detection were 34.1% and 37.1%, cancer detection were 1.2% and 1.9%, and colitis detection were 6.2% and 4.9%, respectively, with no statistically significant differences between the 2 groups (*P* > .05) (Table 3). Table 4 shows the incidence of bowel preparation-related adverse events, including nausea, vomiting, abdominal pain and abdominal fullness. There was not statistical difference between the 2 groups in terms of nausea, vomiting and abdominal pain (*P* > .05). But the 4L-PEG group was significantly higher than the 2L-PEG-CaO group in terms of the rate of abdominal fullness (9.6% vs 6.4%, *P* = .045) and adverse events (33.7% vs 28.5%, *P* = .048).

**Table 3:** Comparison of colonoscopy results.

	2-PEG-CaO (n = 513)	4-PEG (n = 738)	*P* value
Medical results, n (%)			
Normal	238 (46.4)	327 (44.3)	.466
Polyps	53 (10.3)	85 (11.5)	.510
Adenoma	175 (34.1)	274 (37.1)	.274
Cancer	14 (1.2)	6 (1.9)	.313
Colitis	32 (6.2)	36 (4.9)	.297
Others	9 (1.8)	12 (1.6)	.862

CaO = castor oil, PEG = polyethylene glycol.

**Table 4:** Comparison of adverse reactions between the 2 groups.

	2-PEG-CaO (n = 513)	4-PEG (n = 738)	*P* value
Nausea, n (%)	78 (15.2)	106 (14.4)	.679
Vomiting, n (%)	22 (4.3)	38 (5.1)	.484
Abdominal pain, n (%)	48 (9.4)	79 (10.7)	.438
Abdominal fullness, n (%)	33 (6.4)	71 (9.6)	.045*
Total, n (%)	146 (28.5)	249 (33.7)	.048*

CaO = castor oil, PEG = polyethylene glycol.

* *P* < .05.

## 4. Discussion

Early screening for colon cancer is the most effective method to reduce the prevalence and mortality of colorectal cancer. Colonoscopy is an important tool for diagnosing and screening colon lesions, and ideal bowel preparation is an important step to complete the colonoscopy.^[21]^ Besides, the ideal bowel preparation regimen not only requires effective bowel cleansing, but also easy acceptance by the patients.^[22]^

It is well known that the inpatient status has been identified as a risk factor for inadequate bowel preparation.^[23]^ The study found that about 30% of patients had inadequate bowel preparation, which may affect the detection rate of colon polyps and neoplasms and increase the demand for repeat examinations.^[24]^ This may have negative effects on the cost and consumption of healthcare resources, and patients may also have increased hospital stay and costs as a result.

Currently, studies have identified factors affecting bowel cleansing in inpatients.^[7,8]^ Among them, we cannot improve the quality of bowel preparation by changing in patient with complications such as diabetes or the length of hospital stay before colonoscopy. However, bowel cleansing can be improved by modifying the dosing regimen of bowel preparation and the type of bowel preparation. It is worth noting that the main method of high-quality bowel preparation for colonoscopy in outpatients is to adopt a split-dose regimen,^[4,5]^ which is equally widely implemented in clinical practice in inpatients. It is generally accepted that “hard-to-prepare” patients need at least 4L of solution, and possibly more.^[25]^ However, a clinical study suggested that high-volume regimens are no better than low-volume regimens for patients at risk of poor bowel cleansing.^[9]^ According to the current study, low-volume PEG combined with bisacodyl is a more suitable option for patients with a history of colon resection.^[26]^ Similarly, in patients with constipation, reinforced low volume PEG and high volume PEG have similar bowel cleansing effects.^[27]^ These findings are strongly correlated, in large part because low-volume bowel preparation increases patient tolerance and reduces the negative impact of the patient experience.^[28]^

CaO as a safe and effective laxative preparation, its laxative effect is comparable to bisacodyl, the mechanism of CaO cleaning bowel is that after taking CaO, it is decomposed into ricinoleic acid in the duodenum, which stimulates the small intestine and promotes its peristalsis and excretion, thereby inducing a strong laxative effect.^[19,29]^ Studies have found that after orally taken a large dose 50 or 60 mL of CaO, the incidence of adverse reactions such as abdominal cramping, bloating, nausea, vomiting, syncope, and coma increases in patients.^[30,31]^ However, low doses of CaO do not cause serious side effects and it has therefore become widely available as a safe stimulant laxative.^[18,32,33]^ In addition, according to a case report suggesting that pregnant women are prohibited from using CaO.^[34]^ In terms of bowel preparation, a multicenter study suggests that CaO may reduce fluid loading during bowel preparation.^[19]^

In this study, to help inpatients find a better bowel preparation regimen, In response, we compared 2L split PEG plus CaO preparation with the tradition 4L split PEG preparation between bowel cleansing quality and inpatient tolerability. Our results indicated that 2L split PEG solution with CaO is significantly higher than tradition 4L split PEG solution on BBPS, adequate bowel cleansing rate and high-quality bowel preparation. Furthermore, the 2L-PEG-CaO regimen was significantly lower than the 4L-PEG regimen in terms of abdominal distension and overall adverse event rates. The results of this study suggest that 2L split PEG solution with CaO significantly reduces the total amount of oral PEG solution and improves the quality of bowel preparation, while reducing the incidence of abdominal distention and overall adverse events. Although the incidence of abdominal pain was higher in the 2L-PEG-CaO regimen than in the 4L-PEG regimen, the difference was not statistically significant and may be related to the increased bowel motility promoted by CaO. In addition, the medical cost of the 2L-PEG-CaO regimen was also lower than that of the 4L-PEG regimen. Therefore, the 2L-PEG-CaO regimen greatly increases patient satisfaction and compliance. Moreover, the medical cost of the 2L-PEG-CaO regimen is lower than that of the 4L-PEG regimen, which makes it more acceptable to patients.

We have to confess some limitations to our study. First, this is a retrospective study, and the clinical information of the patients is extracted from the medical record system, so the obtained information is prone to bias. In this regard, in the future, our team will conduct randomized controlled clinical trials in the future to verify the conclusions of this study. However, there is an advantage to this study, as the inpatients in this study underwent bowel preparation under full medical supervision, reducing confounding factors associated with poor bowel preparation, such as type of diet before colonoscopy, bowel preparation the experience of preparing and the time to start preparing. Second, while BBBS scores are validated and widely used to evaluate bowel cleansing effects, there is a subjective judgment between each endoscopist, which may indicate a bias in the evaluation of the 2 methods of intestinal preparation. Third, despite our large sample size, this study is still a single-center study, so a larger study is needed to validate and generalize our conclusions.

## 5. Conclusion

The use of 2L split PEG solution with CaO for bowel preparation before colonoscopy in inpatients can improves the quality of bowel preparation and reduces the incidence of adverse events.

## Acknowledgments

This work was supported by gastroenterology center of Changzhou Second People Hospital.

## Author contributions

**Conceptualization:** Jin Huang.

**Data curation:** Ying Fang, Fangfang Feng, Yiming Cheng, Chunyan Huo.

**Formal analysis:** Ying Fang, Fangfang Feng, Yiming Cheng, Chunyan Huo.

**Writing – original draft:** Zhe Xiong.

**Writing – review & editing:** Zhe Xiong.
